# Pediatric Small Bowel Obstruction From a Migratory Trichobezoar

**DOI:** 10.7759/cureus.105118

**Published:** 2026-03-12

**Authors:** Saagar R Patel, Anthony I Zarka

**Affiliations:** 1 Diagnostic Radiology, University of Central Florida College of Medicine, Orlando, USA; 2 Pediatrics, Mayo Clinic Alix School of Medicine, Rochester, USA; 3 Pediatric Radiology, Nemours Children's Health System, Jacksonville, USA

**Keywords:** acute small bowel obstruction, complications, gastric trichobezoar, intussusception, pancreatitis, rapunzel syndrome, small intestine bezoar

## Abstract

Trichobezoars are rare intraluminal masses formed from ingested hair. They are most commonly located in the stomach. When trichobezoars extend from the stomach into the small intestine, it is referred to as "Rapunzel syndrome." One of the rarer complications of trichobezoars is small bowel obstruction due to distal migration. We present a case of a pediatric patient with a large gastrointestinal trichobezoar that migrated into the distal ileum, resulting in a small bowel obstruction. This case emphasizes the importance of radiographic imaging in the diagnosis of trichobezoars and their potential complications.

## Introduction

A bezoar is an intraluminal accumulation of indigestible material within the gastrointestinal tract [1]. Trichobezoars are composed of ingested hair and occur most frequently in pediatric and adolescent females with underlying trichophagia [2]. They are most commonly localized in the stomach [2]. A gastric bezoar that also extends into the small bowel is known as "Rapunzel syndrome" [2].

Complications of trichobezoar include gastric mucosal erosion, ulceration, perforation of the stomach or the small intestine, intussusception, obstructive jaundice, protein-losing enteropathy, pancreatitis, and even death [3]. Transient pancreatitis is a rare possible complication that is thought to result from mechanical irritation and obstruction at the ampulla of Vater [4]. Distal migration of trichobezoars resulting in small bowel obstruction is a rarely reported complication [5].

Typically, all patients with trichobezoars require an exploratory laparotomy for definitive treatment [6]. Surgical intervention is indicated in patients whose trichobezoars are large, causing discomfort or intestinal obstruction [7]. Endoscopy is often unsuccessful for trichobezoar removal because the mass of hair is often too large and dense to remove through the esophagus [6].

We report a rare case demonstrating trichobezoar migration from the stomach into the distal ileum and ascending colon, which resulted in an acute small bowel obstruction.

## Case presentation

A nine-year-old female patient presented to the emergency department with abdominal pain, nausea, and vomiting. Her mother reported intermittent gastrointestinal symptoms over several months prior to presentation. Laboratory evaluation revealed an elevated lipase level of 1,035 U/L. She was diagnosed with pancreatitis and admitted for further management. An ultrasound obtained during hospitalization demonstrated mild biliary ductal dilation. Given her rapid clinical improvement with intravenous fluids and analgesics, she was discharged the following day with plans for further outpatient imaging and follow-up with pediatric gastroenterology.

At her pediatric gastroenterology visit one month later, she reported recurrent episodes of epigastric pain, early satiety, and weight loss since discharge. On physical examination, a right upper quadrant mass was palpated. A CT scan of the abdomen and pelvis was subsequently ordered.

**Figure 1:**
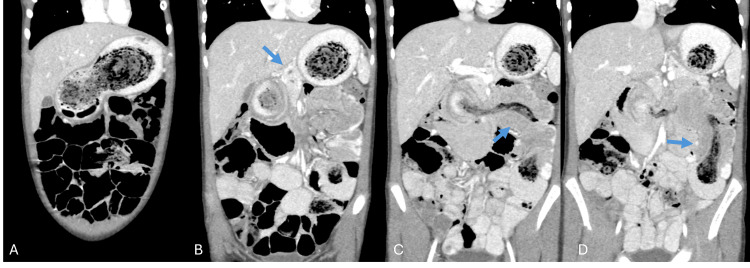
Sequential contrast-enhanced CT images of the abdomen and pelvis demonstrating a large gastric trichobezoar with proximal small bowel extension consistent with Rapunzel syndrome. (A) The coronal image shows a large intragastric trichobezoar. (B) The coronal image demonstrates mass effect on the pancreas with associated pancreatic duct dilation (blue arrow). (C-D) The coronal images demonstrate extension of the trichobezoar into the small bowel complicated by jejunojejunal intussusception (blue arrows). Additional distal extension of the trichobezoar was present but is not shown.

The following day, contrast-enhanced CT of the abdomen and pelvis (Figure 1) demonstrated a large gastric bezoar with elongated extension into the jejunum, consistent with Rapunzel syndrome. The CT also revealed a long-segment jejunojejunal small bowel intussusception. Additionally, the presence of the bezoar resulted in a mass effect on the pancreatic head with associated pancreatic duct dilation.

Following an outpatient consultation with pediatric surgery, an exploratory laparotomy was recommended. However, prior to her scheduled surgery, she re-presented to the emergency department with severe abdominal pain, nausea, and vomiting.

**Figure 2:**
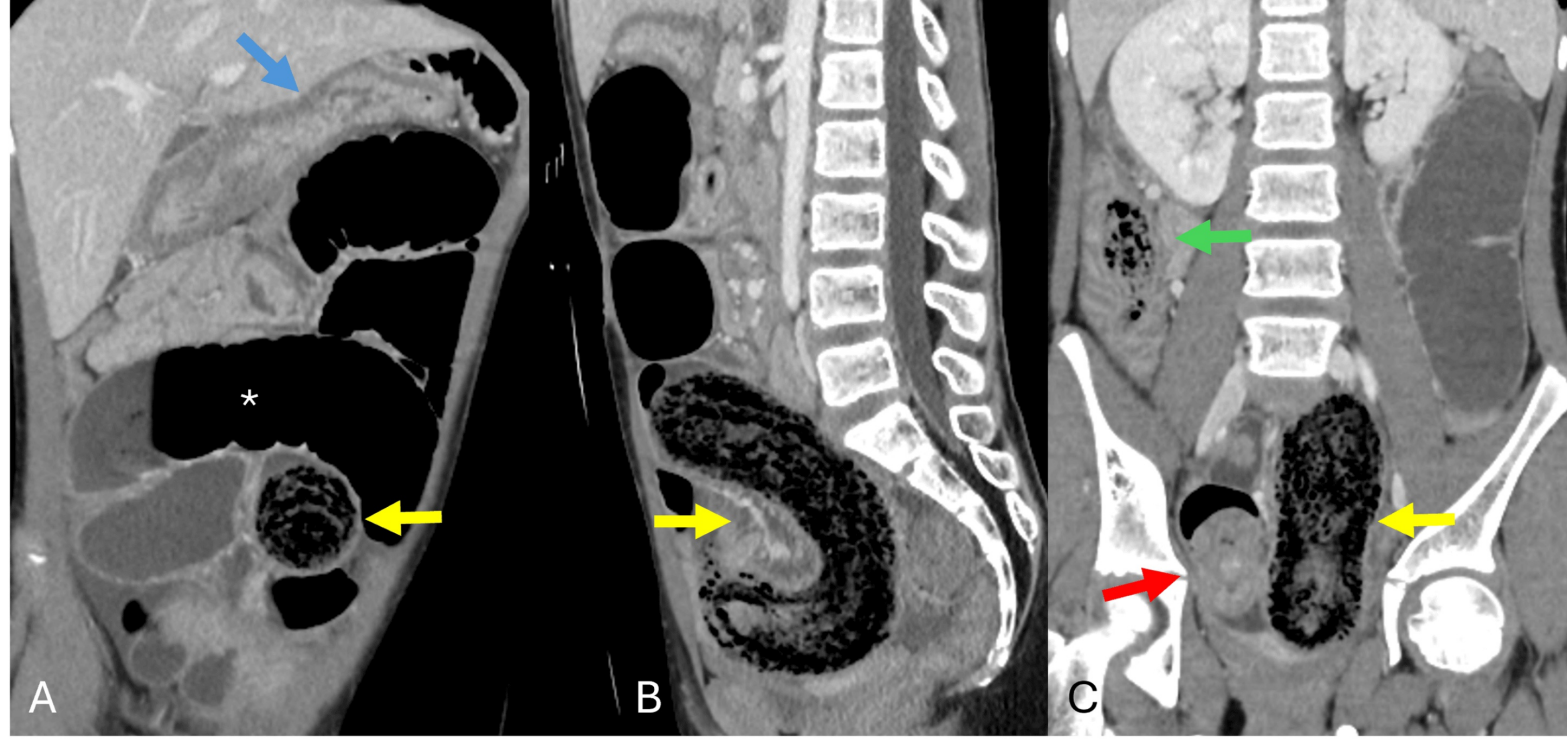
Contrast-enhanced CT images of the abdomen and pelvis demonstrating distal migration of the trichobezoar into the distal ileum with extension into the ascending colon. (A) The coronal image shows an empty stomach (blue arrow) with interval migration of the trichobezoar to the distal ileum (yellow arrow) and associated upstream small bowel dilation (*) consistent with small bowel obstruction. (B) The sagittal image demonstrates the stomach-shaped trichobezoar (yellow arrow) within and obstructing the distal ileum. (C) The coronal image shows the bulk of the trichobezoar (yellow arrow), associated distal ileoileal intussusception (red arrow), and the bezoar “tail” extending into the ascending colon (green arrow).

An abdominal radiograph demonstrated findings consistent with small bowel obstruction. A subsequent contrast-enhanced CT of the abdomen and pelvis (Figure 2) showed that the entire trichobezoar, including the previously identified large gastric component, had migrated to the distal ileum and extended into the cecum.

The patient was taken emergently to the operating room for an exploratory laparotomy. A large, elongated trichobezoar within the distal ileum was identified and removed via enterotomy. She recovered without complication and was discharged four days postoperatively.

## Discussion

Trichobezoars are rare intraluminal masses composed of ingested hair, most commonly occurring in pediatric and adolescent females [8]. They are frequently associated with underlying psychiatric conditions such as trichotillomania, trichophagia, and obsessive-compulsive disorder, in which repetitive hair pulling and ingestion lead to progressive intragastric accumulation [9]. Hair resists digestion and peristalsis due to its smooth surface, allowing entrapment within gastric mucosal folds [3]. Over time, continued ingestion results in enlargement of the trichobezoar. In advanced cases, trichobezoars can extend beyond the pylorus into the small intestine, which is referred to as Rapunzel syndrome [6].

Rapunzel syndrome is an uncommon but clinically significant entity that may lead to multiple complications [3]. Among these, small bowel obstruction is one of the most serious and may occur due to distal migration or elongation of the trichobezoar tail [10,11]. Migration into the distal ileum or colon is particularly rare and may result in mechanical obstruction requiring urgent surgical intervention [6]. Additional reported complications include gastric mucosal erosion, ulceration, perforation of the stomach or small intestine, intussusception, obstructive jaundice, protein-losing enteropathy, pancreatitis, and, in severe cases, death [3]. In this case, distal migration of the bezoar resulted in small bowel obstruction with associated intussusception, highlighting the potential for significant gastrointestinal complications.

Imaging plays a central role in the diagnosis and management of trichobezoars and their complications. CT is considered the imaging modality of choice for diagnosing trichobezoars [5]. CT allows radiologists to identify trichobezoar size, location, and extent of gastrointestinal involvement [5]. Characteristic CT findings include a low-density intraluminal mass containing air bubbles and a mottled gas pattern, and CT is highly sensitive for detecting associated complications such as bowel obstruction, transition points, intussusception, perforation, and pancreaticobiliary involvement.

In this case, CT was instrumental not only in identifying the large gastric trichobezoar and its small bowel extension but also in detecting secondary complications such as small bowel obstruction and intussusception. Trichobezoar-associated pancreatitis is thought to result from mechanical irritation and obstruction at the level of the ampulla of Vater [4]. Recognition of this association is clinically important, as pancreatitis may be an initial presenting feature, albeit rare, that prompts abdominal imaging and subsequent trichobezoar detection.

Management of trichobezoars depends largely on bezoar size and extent. Small, localized gastric bezoars may occasionally be retrieved endoscopically [6]. However, large trichobezoars, especially those seen in Rapunzel syndrome, typically require surgical removal due to their dense composition, extensive length, and the high risk of fragmentation with distal embolization [3]. Exploratory laparotomy remains the most definitive treatment, allowing complete extraction of trichobezoar fragments throughout the gastrointestinal tract [3].

This case underscores the value of CT in the detection and management of trichobezoars. It highlights the potential for distal migration resulting in mechanical small bowel obstruction and emphasizes the importance of early recognition and timely surgical referral in pediatric patients presenting with nonspecific gastrointestinal symptoms and a history suggestive of hair ingestion.

## Conclusions

This case highlights a rare but serious complication of trichobezoars, demonstrating distal migration resulting in acute small bowel obstruction. It underscores the critical role of radiologic imaging in diagnosing and characterizing trichobezoars and their associated complications. CT played a pivotal role in detecting Rapunzel syndrome, delineating the extent of the trichobezoar, and identifying secondary complications. Awareness of these imaging features facilitates prompt surgical management and may improve patient outcomes. Early recognition and timely intervention remain essential to prevent morbidity, particularly in pediatric patients presenting with nonspecific gastrointestinal symptoms and a history suggestive of trichophagia.
